# Dietary parameters in patients with drug allergy: Assessing dietary inflammatory index

**DOI:** 10.1371/journal.pone.0277046

**Published:** 2022-11-03

**Authors:** Eunice Dias de Castro, Sílvia Paredes, Sílvia Pinhão, Josefina R. Cernadas, Laura Ribeiro

**Affiliations:** 1 Allergy and Clinical Immunology Department, Centro Hospitalar Universitário de S. João EPE, Porto, Portugal; 2 MedInUP- Center for Drug Discover and Innovative Medicines, Faculty of Medicine, University of Porto, Porto, Portugal; 3 Public Health and Forensic Sciences and Medical Education Department, Faculty of Medicine, University of Porto, Porto, Portugal; 4 Faculty of Food and Nutrition Sciences, University of Porto, Porto, Portugal; 5 Nutrition Department, Centro Hospitalar Universitário de S. João EPE, Porto, Portugal; 6 Biomedicine Department, Faculty of Medicine, University of Porto, Porto, Portugal; 7 I3S- Instituto de Investigação e Inovação em Saúde, University of Porto, Porto, Portugal; University of Memphis, UNITED STATES

## Abstract

**Background:**

Research on the increasing incidence of allergic diseases evidenced the role of diet as a potential key factor. Diet can modulate the low-grade systemic inflammation related to obesity and several diseases. There are no published data on drug allergy.

**Aim:**

To investigate a potential association between diet, including dietary inflammatory index (DII), and drug allergy. Also, to evaluate correlations between diet and obesity, inflammatory and metabolic parameters in patients with drug allergy.

**Methods:**

Ninety consecutive patients studied for suspected drug allergy were evaluated in terms of dietary parameters, anthropometric measurements, bioimpedance and biochemical analysis. DII was calculated based on information collected from a food frequency questionnaire.

**Results:**

After diagnostic work-up, 39 patients had confirmed drug allergy and 45 excluded, representing the study group and the control group, respectively. The majority (79%) were female, with mean age of 39.58±13.3 years. The 84 subjects revealed an anti-inflammatory diet pattern. No significative difference was found in DII scores between drug allergic patients and controls (-3.37±0.95 vs -3.39±0.86, p = 0.985). However, the patients with drug allergy revealed higher obesity and inflammatory parameters. A significative negative correlation was found between DII and adiponectin levels, in the control group (r = -0.311, p = 0.040). In the patient group, a significative positive correlation was observed between DII and triglycerides (r = 0.359, p = 0.032). No other correlations were found between DII and the assessed parameters. Patients with drug allergy presented a significative higher intake of mono-unsaturated fatty-acids comparing to controls (19.8±3.7 vs 17.8 ± 4.0, p = 0.021). No other statistically significant differences were achieved in dietary parameters, between patients and controls.

**Conclusion:**

The population assessed in this study revealed an anti-inflammatory diet profile. Although we have found in a previous work that the same patients with drug allergy revealed higher obesity and inflammatory parameters, the DII did not allow to distinguish between patients with drug allergy or controls. The DII scores correlated with triglycerides levels in the drug allergy patients and inversely with adiponectin levels in the control group. Larger studies are needed to clarify the potential role of the diet in drug allergy and its outcomes.

## 1 –Introduction

Drug allergic reactions are unpredictable adverse drug reactions (ADR) that are mediated by an immunological mechanism [1, 2]. These reactions are usually classified according to the underlying mechanism [2, 3], but also based on the time of the onset [2]. Several manifestations with different degrees of severity can occur [2–4]. Although it is estimated that drug allergy accounts for less than 10% of all ADR [5], its incidence increased in line with the overall increase in the incidence of allergic diseases [1]. This is especially reported in developed countries and some factors of a westernized lifestyle have emerged as potential contributors, including obesity and changes in dietary pattern ☯5]. In fact, obesity has become epidemic [6] and a major public health problem in the developed world [7, 8]. Much of the disease risk is related to the associated low-grade systemic inflammation [6–10], that has been linked with several chronic conditions and that can be modulated by diet [11–14].

While polyunsaturated fatty acids and antioxidant vitamins have shown a protective role [11, 13, 15, 16], total fat and saturate fat acids revealed pro-inflammatory properties [11, 13, 16].

A dietary pattern approach has been considered advantageous since it considers the combined and interacting effects of nutrients and other food components [12, 17].

The dietary inflammatory index (DII) is a tool, developed by Cavicchia [11], and updated by Shivappa [12], that measures the inflammatory potential of the diet, from maximally anti-inflammatory to maximally pro-inflammatory. The purpose was to be used across diverse population in order to predict inflammatory levels and related health outcomes [13]. It has been used in several studies and chronic diseases [18–24] like metabolic [13] and cardiovascular diseases [19, 20], but also in asthma [22–24].

Research on the increasing incidence of asthma and other allergic diseases evidenced the role of diet as a potential key factor influencing immune homeostasis through a complex interplay between nutrients, their metabolites and immune cell populations [5]. There are no published studies involving patients with drug allergy.

In a previous study [25], we investigated and reported, for the first time, a potential link between obesity and drug allergy. The group of patients with drug allergy revealed significantly higher parameters associated with both global and central obesity and also higher levels of systemic inflammatory markers, than controls. Moreover, body fat percentage (BFP) emerged as a predictor of drug allergy.

The aim of this study was to investigate a potential association between diet, including dietary inflammatory index, and drug allergy.

It was also our objective to evaluate correlations between dietary parameters and obesity, inflammatory and metabolic parameters, in drug allergic patients.

## 2 –Material and methods

To pursuit the proposed aims we developed a prospective, observational, cross-sectional and case-control study.

### Study population

Ninety consecutive adult patients with a suggestive history of drug allergy were prospectively studied in our Department, from September 2017 to October 2018. A complete history based on a validated questionnaire (the drug hypersensitivity questionnaire developed by European Network of Drug Allergy- ENDA) [26] was obtained in the outpatient clinic, during the first clinical assessment. Patients with a suggestive history of drug allergy were referred to a day hospital for diagnostic procedures, as routine in our clinical practice. One hundred of patients that met the inclusion criteria were invited to participate by phone the day before their first visit to the day hospital. If they agreed to participate, an overnight fasting was recommended. The inclusion criteria were: age ≥18 years; history suggestive of drug allergy in the previous 5 years and any suspected drug involved. Patients that did not complete the drug allergy diagnostic protocol or that did not have a conclusive diagnosis at the end of the study, were excluded. The same population were used in our previous work [25] to assess obesity parameters in patients with drug allergy, where the algorithm for patients´ inclusion is outlined.

Social-demographic data were collected.

Leisure-time physical activity was evaluated through two questions about the type of exercise and the time spend on it in a week (number of hours) and classified in intensity categories (sedentary, irregularly active, active and intensely active), considering the 4 categories of the Intensity Physical Activity Questionnaire (IPAQ) classification [27].

The protocol was approved by the Hospital Ethics Committee (CE-66-2016) and written informed consent was obtained from all participants included in the study.

### Drug allergy diagnostic work-up

The diagnostic work-up was performed considering clinical history and according to international guidelines [2], as usual in our clinical practice. The characteristics of the reaction, namely the type (immediate versus non-immediate), clinical manifestations (anaphylaxis, urticaria/angioedema, or maculopapular exanthema, for example) and severity of reaction, are relevant factors to guide diagnostic procedures. These included skin tests, prick and intradermal [28, 29], specific IgE and drug provocation tests (DPT) [30].

The diagnosis of drug allergy was confirmed when skin tests were positive for validated concentrations or when specific provocation test with the suspected drug was positive (group of patients with drug allergy). If all diagnostic procedures were negative, including DPT with the suspected drug, drug allergy was excluded (control group). So, the study randomization was done according to the result of the drug allergy diagnostic work-up.

### Dietary inflammatory index (DII)

The Dietary inflammatory Index (DII) score is a calculated parameter that provides an estimate of the inflammatory potential of the diet. An individual’s diet is considered more pro-inflammatory when the DII score is more positive, while the diet is considered more anti-inflammatory when the DII score is more negative. The DII score was calculated according to Shivappa *et al* [13] and categorizes a list with 45 possible food items, which range from maximally anti-inflammatory to maximally pro-inflammatory potential.

Dietary data were obtained from a 91-item self-administered and semi-quantitative food-frequency questionnaire validated (FFQ) for Portuguese population [31]. This questionnaire was applied indirectly by a nutritionist to all participants allowing to assess the eating habits related to the previous 12 months. Dietary intake estimation was made multiplying the portion size in grams by the multiple fraction of daily frequency intake and by a seasonality variation factor. The conversion, from food to energy and nutrients intake, was performed using The Food Processor Plus program version SQL (ESHA Research, Salem, OR, USA). This database was supplemented with the Portuguese food composition database [32].

The DII score was calculated considering 28 of 45 possible food parameters. Data on β-carotene, eugenol, garlic, ginger, onion, saffron, turmeric, pepper, green/black tea, thyme/oregano, rosemary, flavan-3-ol, flavones, flavonols, flavonones, isoflavones and anthocyanidis, were not included since no information was available for these components in the Food Processor nutritional database.

To derive the individual DII scores, the global average intake (taken from Shivappa et al [13]) was subtracted from the reported daily intake of each nutrient in the FFQ and divided by the standard deviation of the global daily intake, rendering a z-score which was converted into a centered percentile score. This score was then multiplied with an overall inflammatory effect score. All 28 parameters included individually calculated nutrient-specific effect scores were then summed to obtain the global DII score.

### Anthropometric measurements and body composition

Height (to the nearest 0.5 cm) and body weight (to the nearest 0.1 kg) were measured with patients wearing light clothing and no shoes.

Body mass index (BMI) was calculated by dividing weight by squared height. According to the BMI categories defined by World Health Organization guidelines, patients were classified as normal weight (≥18.5 and < 25 kg/m^2^); overweight (≥ 25.0 and <30 kg/m^2^) and obese (≥30.0 kg/m^2^) [33].

Waist circumference (WC) was measured at the level midway between the lowest rib and the iliac crest. Based on International Diabetes Federation (IDF) criteria, central obesity was defined as WC ≥0.80 m in women and ≥0.94 m in men [34, 35]. Hip circumference (HC) was measured at the level of the widest circumference over the great trochanters. Both circumferences were measured with a flexible tap with subjects standing at the end of gentle expiration. The waist-hip ratio (WHR) was calculated [33].

Body composition, including fat mass (Kg and percentage), was assessed by bioimpedance (*Inbody 230*, Body Composition Analyzer; tetra polar 8-point tactile electrodes, BIOSPACE).

### Biochemical analysis

Serum leptin and serum adiponectin concentration were measured with the double antibody sandwich ELISA assay by using an antibody specific for human leptin and for human adiponectin, respectively (Mercodia Leptin ELISA/Mercodia Adiponectin, Mercodia AB, Uppsala, Sweden). Blood samples were obtained after overnight fasting, between 8 and 9 am. After clotting at 4°C, serum was separated by centrifugation and stored at– 70° until assay.

Blood samples for glucose, total cholesterol, high-density lipoprotein (HDL) and triglycerides in serum were obtained, at the same time using anticoagulant-treated tubes and their concentrations determined by standard techniques. The low-density lipoprotein (LDL) levels were calculated according to the manufacturer’s instructions (Roche, Rotkreuz, Switzerland).

### Statistical analysis

The statistical Package for Social Sciences® version 25 was used to analyze data and statistical significance was defined as p < 0.05.

For continuous quantitative variables, the existence of normal distribution was tested through histogram observation, kurtosis and skewness analysis and using the Kolmogorov-Smirnov test. To describe these variables, central tendency measures (mean and median) and dispersion measures (standard-deviation and percentiles 25–75) were used. To compare continuous variables with normal and non-normal distribution between two groups, an Independent Samples t Test and a Mann Whitney test were used, respectively. To compare continuous variables with normal and non-normal distribution between more than two groups, an ANOVA with Bonferroni correction and a Kruskal-Wallis test were used, respectively. For qualitative variables we used absolute numbers and percentages. To analyze differences between groups in categorical variables, the chi-square test and the Fisher exact test were used. Correlations were evaluated using the Pearson and the Spearman correlation tests for continuous symmetrical and asymmetrical variables, respectively.

## 3 –Results

### Study population

Eighty-four patients completed the drug allergy diagnostic work-up with a conclusive result: 39 patients (46%) had the final diagnosis of confirmed drug allergy and in 45 (54%) drug allergy was excluded, representing the study group and the control group, respectively.

Seventy-nine per cent of the patients were female and the mean age was 39.58 ± 13.3 years, range [18 – 77].

The two groups were homogeneous concerning socio-demographic and general clinical characteristics. There were significative differences concerning the drug allergic reactions between the two groups. More details about the sample used in this study are presented in a previous publication from our group [25].

Regarding BMI, 42.2% of patients had normal weight, 36% were overweight, 19.3% obese and 2.4% underweight. According to the IDF criteria, 57.6% of the women and 41.2% of the men presented central obesity. As already described in our previous work [25], patients with drug allergy revealed significantly higher obesity-related parameters (anthropometrics and evaluated by bioimpedance) and also higher systemic inflammatory markers, compared to controls.

### Dietary inflammatory index (DII) and drug allergy

Globally, the 84 subjects revealed an anti-inflammatory diet pattern with a mean DII of -3.38 ± 0.9 (min -4.66, max -0.93).

No significant differences were found in DII scores between patients with confirmed drug allergy and controls (-3.37 ± 0.95 vs -3.39 ± 0.86, p = 0.985).

Analyzing the 39 patients with confirmed drug allergy, no differences were observed in DII scores regarding the clinical manifestations, the type or the severity of the reactions (Table 1).

**Table 1:** Patients with drug allergy: DII scores and characteristics of the reaction.

	DII score	P value
**Type of reaction**		
**Immediate reactions,** Median (IQR)	-3.58 [(-4.03)—(-3.01)]	0.751
**Non-immediate reactions,** Median (IQR)	-3.21 [(-4.23)— (-2.72)]	
**Severity of reaction**		
**Severe reactions,** Median (IQR)	-3.40 [(-4.00)—(-3.05)]	0.975
**Mild/moderate reactions,** Median (IQR)	-3.57 [(-4.11)—(-2.73)]	
**Anaphylaxis**		
**Yes,** Median (IQR)	-3.32 [(-4.03)—(-3.00)]	0.729
**No,** Median (IQR)	-3.64 [(-4.15)—(-2.74)]	
**MPE**		
**Yes,** Median (IQR)	-3.21 [(-4.54)—(-1.60)]	0.894
**No,** Median (IQR)	-3.58 [(-4.00)—(-3.02)]	

IQR- interquartile range, MPE- maculopapular exanthema

### Dietary inflammatory index (DII) and obesity parameters

There were no significant correlations between DII scores and anthropometric measurements or body composition by bioimpedance analyzed as continues variables, neither in the globality of the evaluated individuals, nor in the patient group or in the control group (Table 2).

**Table 2:** Correlations between DII and anthropometric/bioimpedance parameters.

DII	All individuals (n = 84)			Drug allergy group(n = 39)		Control group(n = 45)	
	R value		P value	R value	P value	R value	P value
**BMI (kg/m-** ^**2**^ **)**	0.096		0.387	0.111	0.507	0.086	0.575
**Waist circumference (cm)**	0.070		0.528	0.040	0.813	0.098	0.524
**Hip circumference (cm)**	0.072		0.517	0.117	0.483	0.001	0.997
**Waist/Hip**	-0.012		0.911	-0.038	0.820	0.013	0.932
**Fat mass (kg)**	0.009		0.940	-0.011	0.949	-0.004	0.981
**Body fat percentage (%)**	-0.038		0.739	-0.063	0.711	-0.028	0.854
**Trunk fat mass (kg)**	0.015		0.894	-0.003	0.986	-0.012	0.938
**Trunk fat percentage (%)**	-0.34		0.761	-0.056	0.742	-0.029	0.854
**Trunk lean mass (kg)**	0.126		0.262	0.141	0.404	0.104	0.500
**Fat free mass (%)**	0.115		0.307	0.145	0.391	0.113	0.465
**Lean mass (kg)**	0.122		0.278	0.153	0.365	0.128	0.408

BMI-Body mass index; BMR- Basal metabolism rate; All variables were analyzed as continues

### Dietary inflammatory index (DII) adipokines levels, biochemical and other parameters

With respect to adipokines levels, a significative and negative correlation was found between DII and adiponectin levels, when all the individuals (r = -0.254, p = 0.021) and the control group (r = -0.311, p = 0.040) were considered, but not the drug allergy group (r = -0.225, p = 0.175) (Table 3). Regarding the other assessed parameters, including biochemical parameters, no significant correlations were observed with DII, except for triglycerides in the drug allergic group (r = 0.359, p = 0.032) (Table 3).

**Table 3:** Correlations between DII and adipokines levels, biochemical parameters and other parameters.

DII	All individuals (n = 84)		Drug allergy group (n = 39)		Control Group (n = 45)	
DII	R value	P value	R value	P value	R value	P value
**Leptin (ng/ml)**	-0.107	0.334	-0.078	0.643	-0.157	0.305
**Adiponectin (ng/ml)**	**-0.254**	**0.021**	-0.225	0.175	**-0.311**	**0.040**
**Ratio leptin/adiponectin**	0.006	0.954	0.082	0.626	-0.057	0.060
**Total cholesterol (mg/dl)**	-0.065	0.568	-0.068	0.689	-0.064	0.686
**HDL cholesterol (mg/dl)**	-0.188	0.097	-0.301	0.070	-0.088	0.577
**LDL cholesterol (mg/dl)**	-0.001	0.994	-0.011	0.951	-0.012	0.942
**Triglycerides (mg/dl)**	0.138	0.229	**0.359**	**0.032**	-0.084	0.596
**Glucose (mg/dl)**	0.053	0.643	0.174	0.302	-0.095	0.556
**Systolic BP (mmHg)**	0.025	0.829	-0.051	0.775	0.024	0.880
**Diastolic BP (mmHg)**	0.010	0.932	0.095	0.595	-0.080	0.616

HDL-high-density lipoprotein; LDL- low-density lipoprotein; BP- Blood pressure

### Macronutrients, micronutrients and drug allergy

In Tables 4 and 5 the dietary parameters are described according to macronutrients and micronutrients, respectively. We found that the drug allergic patients presented a significantly higher intake of mono-unsaturated fatty-acids (MUFA) compared to controls (19.8 ± 3.7 vs 17.8 ± 4.0, p = 0.021). No other statistically significant differences were observed.

**Table 4:** Macronutrients intake and drug allergy diagnosis.

	Confirmed drug allergy (N = 38)	Excluded drug allergy (N = 45)	P value
**Calories (kcal)**, Mean (SD)	2457.3 (648.7)	2488.7 (550.7)	0.812
**Protein (g),** Mean (SD)	111.2 (32.4)	113.6 (25.1)	0.697
**Carbohydrates (g),** Mean (SD)	266.8 (74.8)	284.6 (79.2)	0.298
**Fibre (g),** Mean (SD)	31.0 (11.7)	31.2 (11.3)	0.914
**Total fat (g),** Mean (SD)	106.4 (31.5)	101.3 (26.1)	0.418
**Cholesterol (mg),** Mean (SD)	349.3 (109.8)	338.1 (92.3)	0.616
**Linolenic acid(g),** Median (IQR)	1.4 (1.1–1.7)	1.3 (1.1–1.8)	0.877
**Trans fat (g),** Median (IQR)	0.9 (0.7–1.2)	0.9 (0.7–1.1)	0.844
**Sodium (mg),** Mean (SD)	2112.3 (696.0)	2228.5 (608.9)	0.419
**Water (%),** Mean (SD)	1573.5 (515.4)	1626.8 (461.9)	0.621
**Alcohol (g),** Median (IQR)	1.7 (0.0–5.2)	1.7 (0.0–10.9)	0.464
**Caffeine (mg),** Median (IQR)	81.3 (31.4–105.1)	78.8 (19.2–86.4)	0.187
**Protein (TEV),** Mean (SD)	18.0 (2.0)	18.3 (2.2)	0.477
**Carbohydrates (TEV),** Mean (SD)	43.7 (7.2)	45.5 (5.5)	0.191
**Simple sugars (TEV),** Mean (SD)	18.1 (4.8)	19.4 (4.3)	0.178
**Total fat (TEV),** Mean (SD)	38.9 (5.3)	36.6 (5.5)	0.060
**Polyunsaturated fat (TEV),** Mean (SD)	6.5 (0.9)	6.3 (1.2)	0.695
**Omega-3 (TEV),** Mean (SD)	0.6 (0.1)	0.6 (0.1)	0.593
**Omega-6 (TEV),** Mean (SD)	5.2 (1.0)	5.1 (1.2)	0.558
**Saturated fat (TEV),** Mean (SD)	9.6 (1.7)	9.5 (1.5)	0.820
**Linoleic acid (TEV),** Mean (SD)	5.0 (1.0)	4.9 (1.2)	0.523
**Monounsaturated fat (TEV),** Mean (SD)	**19.8 (3.7)**	**17.8 (4.0)**	**0.021**
**Linolenic acid(TEV),** Median (IQR)	12.9 (9.9–15.4)	12.0 (10.0–16.0)	0.913
**Trans fat (TEV),** Median (IQR)	0.3 (0.3–0.5)	0.3 (0.3–0.4)	0.777
**Ethanol (TEV),** Median (IQR)	11.6 (0.0–36.2)	11.6 (0.0–76.3)	0.409

SD- standard deviation; IQR- interquartile range; TEV-total energy value

**Table 5:** Micronutrients intake and drug allergy diagnosis.

	Confirmed drug allergy (N = 38)	Excluded drug allergy (N = 45)	P value
**Vitamin A (RE)**, Mean (SD)	2313.2 (1183.1)	2483.5 (1342.0)	0.485
**Niacin B3 (mg),** Mean (SD)	26.2 (7.3)	28.6 (8.6)	0.625
**Folate (mcg),** Mean (SD)	418.7 (171.2)	420.4 (177.0)	0.890
**Iron (mg),** Mean (SD)	17.7 (4.9)	18.8 (6.2)	0.371
**Magnesium (mg),** Mean (SD)	418.4 (147.6)	408.6 (120.8)	0.166
**Potassium (mg),** Mean (SD)	4009.5 (1287.4)	4194.5 (1092.6)	0.496
**Selenium (mg),** Mean (SD)	121.1 (40.1)	117.1 (33.9)	0.173
**Zinc (mg),** Mean (SD)	13.5 (4.3)	14.0 (3.9)	0.231
**Vitamin D (mcg),** Mean (SD)	4.7 (2.0)	5.1 (2.0)	0.919
**Vitamin E (mg),** Mean (SD)	14.4 (4.4)	14.0 (4.4)	0.936
**Thiamine B1 (mg),** Median (IQR)	1.7 (1.5–2.1)	1.8 (1.5–2.2)	0.285
**Riboflavin B2 (mg),** Median (IQR)	2.1 (1.7–2.6)	2.2 (1.8–2.7)	0.344
**Vitamin B6 (mg),** Median (IQR)	2.3 (2.0–2.9)	2.6 (2.2–3.3)	0.091
**Retinol (RE)**, Median (IQR)	374.8 (257.6–762.2)	329.5 (206.4–803.6)	0.694
**Vitamin B12 (mcg),** Median (IQR)	10.0 (7.7–13.3)	10.1 (7.5–12.3)	0.920
**Vitamin C (mg),** Median (IQR)	156.1 (114.0–230.0)	170.0 (118.5–245.1)	0.590
**Vitamin K (mcg),** Median (IQR)	13.0 (11.0–20.7)	13.6 (11.0–20.8)	0.923
**Calcium (mg),** Median (IQR)	919.4 (766.9–1237.3)	932.1 (838.6–1284.0)	0.609
**Iodine (mcg),** Median (IQR)	70.7 (33.6–99.5)	77.1 (42.8–102.9)	0.577

SD- standard deviation; IQR- interquartile range; RE- retinol equivalent (1RE = 1 mcg Vitamin A)

### Physical activity and obesity, biochemical and other parameters

In terms of physical activity, the majority of individuals (48.8%) were sedentary, 26.2% irregularly active, 9.5% active and 14.3% intensely active. In the group of patients with drug allergy, 21 (53.8%) were classified as sedentary, 9 (23.1%) irregularly active, 4 (10.3%) active and 4 (10.3%) intensely active, while 8 controls (17.8%) were intensely active, 4 (8.9%) active, 13 (28.9%) irregularly active and 20 (44.4%) sedentary.

No differences with statistical significance were obtained between the 4 categories of physical activity regarding anthropometric/ bioimpedance, inflammatory and biochemical parameters assessed or with the DII scores.

## 4 –Discussion

To our knowledge, this is the first study applying the DII score in the assessment of patients with drug allergy. We found a slightly higher DII score in patients with drug allergy compared to controls but not achieving statistical significance. Nonetheless, the former group revealed significantly higher obesity parameters and leptin levels, as detailed in our previous work [25]. This result can be in part related to the general dietary pattern of our sample, but also to the sample size. Interestingly, the globality of the individuals of our study presented an anti-inflammatory DII (DII = -3.38), suggesting a Mediterranean diet pattern [6]. This DII score is considerably more anti-inflammatory than the few studies involving patients with asthma, performed in Australia [24] (DII asthma group = −1.40±0.2; controls = −1.86±0.4, p = 0.04), in Portugal [36] (0.1 ± 1.5) and in EUA [23] (DII scores revealed a pro-inflammatory diet in all evaluated groups). Other studies carried out in our country also showed higher DII scores, particularly studies involving children [36, 37] or adolescents [19], but also adults (-0.35 ± 1.88) [38]. These differences might also reflect regional and age differences in dietary patterns.

In our study, no significant correlations were found between DII scores and obesity parameters assessed by anthropometric measurements or by bioimpedance. In very large sample studies, individuals with higher pro-inflammatory diets assessed by DII presented higher obesity parameters, such as BMI [20, 39, 40] and especially waist circumference [20]. With these results the authors discuss that a pro-inflammatory diet might contribute to increase or maintain obesity, especially abdominal obesity, in a population mostly overweight or obese [20]. It is well established that the link between obesity and diet is not exclusively related to the balance between energy intake and expenditure [6], but also with the inflammatory pattern of the diet and its potential to modulate the low-grade chronic inflammation state associated with obesity [12–15]. Several studies investigated and found associations between DII scores and inflammatory systemic markers, as CRP [14, 42], TNF-alfa [22, 42], IL6 [19, 23], C3 [19, 42], and also with biochemical parameters as triglycerides [41, 42], and other lipoproteins [41].

Within the inflammatory and biochemical parameters assessed in our study, we found a negative correlation between the DII scores and adiponectin levels, when all the individuals or only the control group were considered. This correlation was not present in the drug allergy group. Adiponectin is an anti-inflammatory adipokine that is downregulated in the obesity status [43]. A negative correlation with DII scores means that patients with lower DII scores or more anti-inflammatory diet, have higher adiponectin serum levels which is in accordance with the adiponectin role and with other studies [41]. Moreover, this correlation was observed in the control group, with less obesity parameters and leptin levels [25], but not in the drug allergic patients. We also observed a positive correlation between DII scores and triglycerides levels exclusively in the drug allergic group, illustrating that patients with a less anti-inflammatory diet have higher triglycerides levels. Also, this correlation was only present in the group with higher obesity parameters and systemic inflammatory parameters [25].

With respect to individual macro and micronutrients and other food components, we did not find any association with drug allergy, except for MUFAs, with higher intake in this group compared to controls. This is an unexpected result, as MUFAs have globally shown anti-inflammatory effects [44]. Moreover, this result does not take into account the whole diet and the interaction between its components, and a dietary pattern approach is more appropriate [13, 18, 44, 45]. Although, Heinrich *et al*, in 2001, used the European Community Respiratory Health Survey (ECRHS) that included 3,872 subjects, and concluded that a high intake of MUFAs might promote the development of allergic sensitization [46].

In terms of the potential role of the diet and its components in the development of allergic diseases and its outcomes, research is focused not only in dietary patterns [23, 24, 36, 43, 45], but also in individual nutrients and food components^,^ [47–49] and their potential influence throughout life, including in fetal life [50]. Although some results are inconsistent, there is evidence that the Mediterranean diet pattern has a protective role [6, 44, 45, 47] and a Western diet pattern a deleterious one [6, 44]. This is mostly attributed to the high content in antioxidants, fibers, polyunsaturated fatty-acids omega-3 (w3-PUFAs) and MUFAs of the Mediterranean diet, combined with low amounts of saturated fatty-acids (SFAs) [6, 44, 45, 47]. Conversely, the Western diet is a high caloric diet, rich in SAFs and w-6 PUFAs, and with low intake of w-3 PUFAs, fiber and antioxidants [6, 44, 45, 47]. The role of individual nutrients and other food components is not yet clearly established in allergic diseases, although several have revealed potential effects. The ratio w3-PUFAs/w-6 PUFAs might be important [44, 45, 49], while fruits and vegetables have revealed to have the most consistent protective effects [42, 44, 47, 48], probably because they are rich in antioxidants such as vitamin C, E and β-carotene and also in flavonoids, isoflavonoids and polyphenolic compounds [45, 48]. On the other hand, vitamin A and D deficiency might influence the development of allergy and its outcomes [6, 47].

Physical activity was also evaluated in this study, as exercise has demonstrated an effect on total and abdominal adiposity [51], serum adipokines [52] and biochemical parameters [53].

No statistically significant differences were found between the 4 categories of physical activity regarding anthropometric/ bioimpedance, inflammatory and biochemical parameters evaluated, probably due to the reduced number of individuals in each category. We also analyzed a potential association between physical activity and DII scores, as both may reflect a certain type of lifestyle, but also here the sample size might have influenced the absence of a significant association.

The global anti-inflammatory diet pattern of our sample might represent a constraint to the results of our study. Other limitations of our study also deserve consideration. First, the sample size also did not allow the analysis according to the standardized terciles or quartiles as seen in other studies. Second, 14 nutrients/components were not included in the DII assessment, as no data was available in the Portuguese food composition database. Third, dietary habits were collected using self-reported information from FFQs and based on patients recall from the last 12 months. However, the use of a validated FFQ is considered an appropriate tool for this purpose and permits seasonality intake. This is not possible with 24h-Food questionnaires but, on the other hand, these questionnaires are more specific and do not have recall bias. Fourth, a trained nutritionist assisted the participants in our study. This fact could avoid some inaccuracies, but could also influence patients´ answers, as they tend to respond according to social desirability [54]. Finally, the method used for the evaluation of physical activity was not based on objective measures and the question of the social desirability may also have influenced the patients´ answers.

On the other hand, the prospective design, the randomization, the high number of dietary parameters evaluated, the assessment of a considerable number of potential confounders and the complete drug-allergy work-up were relevant in our study.

## 5- Conclusions

Globally the population assessed in this study revealed an anti-inflammatory diet profile. Although, we have found in a previous work that the same patients with drug allergy revealed higher obesity and inflammatory parameters, here the DII did not allow to distinguish between patients with drug allergy and controls. However, the DII scores correlated with triglycerides levels in drug allergic patients and inversely with adiponectin levels in the control group.

Larger studies are needed to clarify the potential role of the diet in drug allergy and its outcomes.

## Supporting information

S1 File (SAV)

## Abbreviations

DII: dietary inflammatory index
ADR: adverse drug reaction
ENDA: European Network of Drug Allergy
IPAQ: Intensity Physical Activity Questionnaire
DPT: drug provocation test
FFQ: food frequency questionnaire
BMI: body mass index
WC: waist circumference
IDF: International Diabetes Federation
HP: hip circumference
WHR: waist-hip ratio
HDL: high-density lipoprotein
LDL: low-density lipoprotein
BFP: body fat percentage
MUFA: mono-unsaturated fatty acid
PUFA: polyunsaturated fatty acid
SFA: saturated fatty-acid
SD: standard deviation
IQR: interquartile range
BP: blood pressure

## References

[1] Broyles AD, Banerji A, Castells M., 2020. Practical Guidance for the Evaluation and Management of Drug Hypersensitivity: General Concepts. *J Allergy Clin Immunol Pract* 8 (9): S3–S15. Epub 2020 Aug 11. doi: 10.1016/j.jaip.2020.08.002 . 32791249

[2] Demoly P, Adkinson NF, Brockow K, Castells M, Chiriac AM, Greenberger PA et al., 2014. International Consensus on drug allergy. *Allergy* 69:420–37. doi: 10.1111/all.12350 . 24697291

[3] Schnyder B, Pichler WJ., 2009. Mechanisms of Drug-Induced Allergy. *Mayo Clin Proc*; 84 (3):268–272. doi: 10.1016/S0025-6196(11)61145-2 ; PMCID: PMC2664605. 19252115 PMC2664605

[4] Pichler WJ, Naisbitt DJ, Kevin Park B., 2011. Immune pathomechanism of drug hypersensitivity reactions. *J Allergy Clin Immunol* 127, 3: S74–S81. doi: 10.1016/j.jaci.2010.11.048 . 21354503

[5] Thong BY, Tan TC., 2011. Epidemiology and risk factors for drug allergy. *Br J Clin Pharmacol* 71:684–700. doi: 10.1111/j.1365-2125.2010.03774.x ; PMCID: PMC3093074. 21480948 PMC3093074

[6] Julia V, Macia L, Dombrowicz D., 2015. The impact of diet on asthma and allergic diseases. *Nat Rev Immunol* 15, 308–322. doi: 10.1038/nri3830 . 25907459

[7] Saltiel AR, Olefsky JM., 2017. Inflammatory mechanisms linking obesity and metabolic disease. *J Clin Invest* 127 (1): 1–4. Epub 2017 Jan 3 doi: 10.1172/JCI92035 28045402 PMC5199709

[8] Shin J, Syme C, Wang D, Richer L, Pike GB, Gaudet D et al., 2019. Novel genetic locus of visceral fat and systemic inflammation. *J Clin Endocrinol Metab* 104 (9): 3735–3742. doi: 10.1210/jc.2018-02656 ; PMCID: PMC6642667 30942860 PMC6642667

[9] Grant RW, Dixit VD., 2015. Adipose Tissue as an Immunological Organ. *Obesity* 23(3):512–8. doi: 10.1002/oby.21003 25612251 PMC4340740

[10] Liu R, Nikolajczky BS., 2019. Tissue Immune Cells Fuel Obesity-Associated Inflammation in Adipose Tissue and Beyond. *Frontiers in Immunology* 10: 1587. doi: 10.3389/fimmu.2019.01587 31379820 PMC6653202

[11] Longo M, Zatterale F, Naderi J, Parrillo L, Formisano P, Raciti GA et al., 2019. Adipose tissue dysfunction as determinant of obesity-associated metabolic complications. *Int Jo Mol Sci* 20, 2358. doi: 10.3390/ijms20092358 31085992 PMC6539070

[12] Cavicchia PP, Steck SE, Hurley TG, Hussey JR, Ma Y, Ockene IS et al., 2009. A new dietary inflammatory index predicts interval changes in serum high-sensitivity C-reactive protein. *J Nutr* 139(12):2365–72. doi: 10.3945/jn.109.114025 ; PMCID: PMC2777480. 19864399 PMC2777480

[13] Shivappa N, Steck SE, Hurley TG, Hussey JR, Hebert JR., 2014. Designing and developing a literature-derived, population-based dietary inflammatory index. *Pub Health Nutr* 17 (8):1689–96. doi: 10.1017/S1368980013002115 ; PMCID: PMC3925198. 23941862 PMC3925198

[14] Shivappa N, Steck SE, Hurley TG, Hussey JR, Ma Y, Ockene IS et al., 2014. A population-based dietary inflammatory index predicts levels of C-reactive protein in the Seasonal Variation of Blood Cholesterol Study (SEASONS). *Pub Health Nutr*. 2014;17 (8):1825–33. Epub 2013 Oct 10. doi: 10.1017/S1368980013002565 ; PMCID: PMC3983179. 24107546 PMC3983179

[15] Phillips CM, Chen L, Heude B, Bernard JY, Harvey NC, Duijts L et al.,2019. Dietary Inflammatory Index and Non-Communicable Disease Risk: A Narrative Review. *Nutrients* 11, 1873. doi: 10.3390/nu11081873 31408965 PMC6722630

[16] Connaughton RM, McMorrow AM, McGillicuddy FC, Lithander FE, Roche HM., (2016). Impact of anti-inflammatory nutrients on obesity- associated metabolic-inflammation from childhood through to adulthood. *Proc Nutr Soc* 1–10. Epub 2016 Mar 3. doi: 10.1017/S0029665116000070 26934951

[17] Calder PC, Ahluwalia N, Brouns F, Buetler T, Clement K, Cunningham K et al., 2011. Dietary factors and low-grade inflammation in relation to overweight and obesity. *Br J Nutr* 106 (Sup 3):S5–78. doi: 10.1017/S0007114511005460 . 22133051

[18] Barbaresko J, Koch M, Schulze MB, Nöthlings U., 2013. Dietary pattern analysis and biomarkers of low-grade inflammation: a systematic literature review. *Nutr Ver* 71(8):511–27. Epub 2013 Jun 13. doi: 10.1111/nure.12035 . 23865797

[19] Almeida-de-Souza J, Santos R, Barros R, Abreu S, Moreira C, Lopes C et al., 2018. Dietary inflammatory index and inflammatory biomarkers in adolescents from LabMed physical activity study. *Eur J Clin Nutr*. 2018;72(5):710–719. Epub 2017 Dec 26. doi: 10.1038/s41430-017-0013-x . 29277838

[20] Ruiz-Canela M, Zazpe I, Shivappa N, Hebert JR, Sanchez-Tainta A, Corella D et al., 2015. Dietary inflammatory index and anthropometric measures of obesity in a population sample at high cardiovascular risk from the PREDIMED (PREvencion con DIeta MEDiterranea) trial. *Br J Nutr* 113(6):984–95. Epub 2015 Feb 27. doi: 10.1017/S0007114514004401 ; PMCID: PMC4870040. 25720588 PMC4870040

[21] Garcia-Arellano A, Ramallal R, Ruiz-Canela M, Salas-Salvado J, Corella D, Shivappa N et al., 2015. Dietary Inflammatory Index and Incidence of Cardiovascular Disease in the PREDIMED Study. *Nutrients* 7(6):4124–38. doi: 10.3390/nu7064124 ; PMCID: PMC4488776. 26035241 PMC4488776

[22] Shivappa N, Hebert J, Marcos A, Diaz L-E, Gomez S, Nova E et al., 2017. Association between dietary inflammatory index and inflammatory markers in the HELENA. *Mol Nutr Food Res* 61 (6). Epub 2017 Feb 22. doi: 10.1002/mnfr.201600707 ; PMCID: PMC5517083. 27981781 PMC5517083

[23] Han YY, Forno E, Shivappa N, Wirth MD, Hebert JR, Celedon JC., 2018. The dietary inflammatory index and current wheeze among children and adults in the United States. *J Allergy Clin Immunol Pract*. 6(3):834–841.e2. doi: 10.1016/j.jaip.2017.12.029 29426751 PMC5948124

[24] Wood LG, Shivappa N, Berthon BS, Gibson PG, Hebert JR., 2015. Dietary inflammatory index is related to asthma risk, lung function and systemic inflammation in asthma. *Clin Exp Allergy*. 45(1): 177–183. doi: 10.1111/cea.12323 ; PMCID: PMC4190104. 24708388 PMC4190104

[25] Dias de Castro E, Pinhão S, Paredes S. Cernadas JR, Ribeiro JRL., 2021. Obesity markers in patients with drug allergy and body fat as a predictor. *Ann Allergy Asthma Immunol* 127 (1):100–108. Epub 2021 Mar 23. doi: 10.1016/j.anai.2021.03.014 . 33771681

[26] Demoly P, Kropf R, Bircher A, Pichler WJ., 1999. Drug hypersensitivity questionnaire (ENDA-EAACI). *Allergy* 54: 999–1003. doi: 10.1034/j.1398-9995.1999.00247.x . 10505466

[27] Craig CL, Marshall AL, Sjostrom M, Bauman AE, Botth ML, Ainsworth BE et al., Pratt M, Ekelund U, 2003. International Physical Activity Questionnaire: 12-Country Reliability and Validity, Medicine & Science in Sports & Exercise: August 2003—Volume 35—Issue 8—p 1381–1395. doi: 10.1249/01.MSS.0000078924.61453.FB 12900694

[28] A Brockow K, Romano A, Blanca M, Ring J, Pichler W, Demoly P., 2002. General considerations for skin test procedures in the diagnosis of drug hypersensitivity. *Allergy* 57:45–51. 11991289

[29] Brokow K, Garvey LH, Aberer W, Atanaskovic-Markovic M, Barbaud A, Bilo MB et al; ENDA/EAACI Drug Allergy Interest Group., 2013. Skin test concentrations for systemically administered drugs. *Allergy* 68: 702–712. Epub 2013 Apr 25. doi: 10.1111/all.12142 . 23617635

[30] Aberer W, Bircher A, Romano A, Blanca M, Campi P, Fernadez J et al. European Network for Drug Allergy (ENDA); EAACI interest group on drug hypersensitivity., 2003. Drug provocation testing in the diagnosis of drug hypersensitivity reactions: general considerations. *Allergy* 58(9):854–63. doi: 10.1034/j.1398-9995.2003.00279.x . 12911412

[31] Lopes C, Aro A, Azevedo A, Ramos E, Barros H et al., 2007. Intake and adipose tissue composition of fatty acids and risk of myocardial infarction in a male Portuguese community sample. *J Am Diet Assoc*. 107(2):276–86. doi: 10.1016/j.jada.2006.11.008 . 17258965

[32] Martins I, Porto A, Luísa O., 2006. Table Composition of Foods. Lisbon, Portugal: National Institute of Health Dr Ricardo Jorge.

[33] WHO Expert Committee. Physical Status: Use and Interpretation of Anthropometry. WHO Technical Report Series 1995; 854. 8594834

[34] Klein S, Allison DB, Heymsfield SB, Kelley DE, Leibel RL, Nonas C et al., 2007. Waist circumference and cardiometabolic risk: a consensus statement from Shaping America’s Health: Association for Weight Management and Obesity Prevention; NAASO, The Obesity Society; the American Society for Nutrition; and the American Diabetes Association. *Am J Clin Nutr*. 85:1197–1202. doi: 10.1093/ajcn/85.5.1197 . 17490953

[35] Alberti K, Eckel R, Grundy S, Zimmet PZ, Cleeman JI, Donato KA et al., 2009. Harmonizing the Metabolic Syndrome. A Joint Interim Statement of the International Diabetes Federation Task Force on Epidemiology and Prevention; National Heart, Lung, and Blood Institute; American Heart Association; World Heart Federation; International Atherosclerosis Society; and International Association for the Study of Obesity. *Circulation* 120 (16):1640–1645. Epub 2009 Oct 5. doi: 10.1161/CIRCULATIONAHA.109.192644 . 19805654

[36] Castro Mendes F, Paciência I, Rufo JC, Silva D, Cunha P, Farraia M et al., 2020. The inflammatory potential of diet impacts the association between air pollution and childhood asthma. *Pediatr Allergy Immunol* 31:290–296. Epub 2020 Jan 14. doi: 10.1111/pai.13185 . 31816137

[37] Machado V, Botelho J, Viana J, Pereira P, Lopes L.B, Proença L et al., 2021. Association between Dietary Inflammatory Index and Periodontitis: A Cross-Sectional and Mediation Analysis. *Nutrients* 13:1194. doi: 10.3390/nu13041194 33916342 PMC8066166

[38] Esteban-Cornejo I, Mota J, Abreu S, Pizarro AN, Santos MP., (2018). Dietary inflammatory index and academic performance in children. *Public Health Nutrition* 21(17):1–5. Epub 2018 Aug 8. doi: 10.1017/S1368980018001994 . 30088468 PMC10260789

[39] Kord Varkaneh H, Fatahi S, Tajik S, Rahmani J, Zarezadeh, Shab-Bidar S(2018). Dietary inflammatory index in relation to obesity and body mass index: A meta-analysis. *Nutr*. *Food Sci* 48(5): 702–721. doi: 10.11o8/NFS-09-2017-0203

[40] Garcia-Arellano A, Martinez-Gonzalez M A., Ramallal R, Salas-Salvadó J, Hebert J.R, Corella D, et al.,2019. Dietary inflammatory index and all-cause mortality in large cohorts: The SUN and PREDIMED studies. *Clin. Nutr*. 38 (3): 1221–1231. Epub 2018 May 24. doi: 10.1016/j.clnu.2018.05.003 . 30651193

[41] Phillips CM, Shivappa N, Hébert JR, Perry., 2018. Dietary Inflammatory Index and Biomarkers of Lipoprotein Metabolism, Inflammation and Glucose Homeostasis in Adults. *Nutrients* 10(8):1033. doi: 10.3390/nu10081033 30096775 PMC6115860

[42] Young Oh H, Lee S-Y, Yoon J, Cho H-J, Kim Y-H, Suh DI, et al., 2020. Vegetable dietary pattern may protect mild and persistent allergic rhinitis phenotype depending on genetic risk in school children. *Pediatr Allergy Immunol*. 31(8): 920–929. Epub 2020 Jul 9. doi: 10.1111/pai.13308 . 32524629

[43] Graßmann S, Wirsching J, Eichelmann F, Aleksandrova K., 2017. Association Between Peripheral Adipokines and Inflammation Markers: A Systematic Review and Meta-Analysis. *Obesity* 25 (10), 1776–1785. Epub 2017 Aug 20. doi: 10.1002/oby.21945 . 28834421

[44] Guilleminault L, Williams EJ, Scott HA, Berthon BS, Jensen M, Wood LG., 2017. Diet and Asthma: Is it time to adapt our message? *Nutrients* 9 (11): 1127. doi: 10.3390/nu9111227 ; PMCID: PMC5707699. 29117118 PMC5707699

[45] Garcia-Marcos L, Castro-Rodriguez JA, Weinmayr G, Panagiotakos DB, Priftis KN, Nagel G., 2013. Influence of Mediterranean diet on asthma in children: A systematic review and meta-analysis. *Pediatr*. *Allergy Immunol* 24 (4), 330–338. Epub 2013 Apr 11. doi: 10.1111/pai.12071 . 23578354

[46] Heinrich J, Hölscher B, Bolte G, Winkler G., 2001. Allergic sensitization and diet: ecological analysis in selected European cities. *Eur Resp J* 17: 395–402. doi: 10.1183/09031936.01.17303950 . 11405517

[47] Nurmatov U, Devereux G, Sheikh A., 2011. Nutrients and foods for the primary prevention of asthma and allergy: systematic review and meta-analysis. *J Allergy Clin Immunol* 127(3): 724–33. Epub 2010 Dec 24. doi: 10.1016/j.jaci.2010.11.001 . 21185068

[48] Hosseini B, Berthon BS, Wark P, Wood LG., 2017. Effects of fruit and vegetable consumption on risk of asthma, wheezing and immune responses: A systematic review and meta-analysis. *Nutrients* 9 (4): 341. doi: 10.3390/nu9040341 ; PMCID: PMC5409680 28353635 PMC5409680

[49] Miyata J, Arita M., 2015. Role of omega-3 fatty acids and their metabolites in asthma and allergic diseases *Allergol Int* 64 (1): 27–34. Epub 2014 Oct 27. doi: 10.1016/j.alit.2014.08.003 . 25572556

[50] Beckhaus AA, Garcia-Marcos L, Forno E, Pacheco-Gonzalez RM, Celedón JC, Castro-Rodriguez JÁ., 2015. Maternal nutrition during pregnancy and risk of asthma, wheeze and atopic diseases during childhood: a systematic review and meta-analysis. *Allergy* 70 (12): 1588–1604. Epub 2015 Sep 21. doi: 10.1111/all.12729 . 26296633

[51] Brennan AM, Day AG, Cowan TE, Clarke GJ, Lamarche B, Ross R., 2020. Individual Response to Standardized Exercise: Total and Abdominal Adipose Tissue. *Med Sci Sports Exerc*. 52(2):490–497. doi: 10.1249/MSS.0000000000002140 . 31479006

[52] Yu N, Ruan Y, Gao X, Sun J., 2017. Systematic Review and Meta-Analysis of Randomized, Controlled Trials on Effect of Exercise on Serum Leptin and Adiponectin in Overweight and Obese Individuals. *Horm Metab Res* 49(3):164–173. Epub 2017 Mar 1. doi: 10.1055/s-0042-121605 . 28249299

[53] Fernández-García JC, Muñoz-Garach A, Martínez-González MÁ, Salas-Salvado J, Corella D, Hernáez Á et al., 2020. Association between lifestyle and hypertriglyceridemic waist phonotype in the PREDIMED-Plus Study. *Obesity* 2020; 28 (3): 537–543. doi: 10.1002/oby.22728 . 32090511

[54] Poínhos R, Correia F, Faneca M, Ferreira J, Gonçalves C, Pinhão S et al., 2008. Desejabilidade social e barreiras ao cumprimento da terapêutica dietética em mulheres com excesso de peso. *Acta Med Port* 21(3):221–228. 18674414

